# Supplementary material for: Risk factors associated with prolonged viral clearance in patients with a refractory course of COVID-19: a retrospective study
Source: PeerJ. 2021 Nov 23;9:e12535. doi: 10.7717/peerj.12535 (PMC8621710; doi:10.7717/peerj.12535)
Supplement: Supplemental Information 3 [file peerj-09-12535-s003.docx]

**Supplementary Material**

**Supplementary Table S1** Sensitivity analysis of baseline characteristics between patients included in regression analysis and the overall cohort

| **Baseline Characteristics** | **Overall cohort**  **(n=65)** | **Patients included in regression analysis (n=35)** | **p value** |
| --- | --- | --- | --- |
| **Age (years)** |  |  |  |
| ≤60 | 43 (66.2) | 20 (57.1) | 0.373 |
| >60 | 22 (33.8) | 15 (42.9) |  |
| **Male** | 40 (61.5) | 19 (54.3) | 0.482 |
| **Reasons of admission** |  |  | 0.912 |
| Recurrence of symptoms | 3 (4.6) | 1 (2.9) |  |
| Possible reinfection with positive nucleic acid testing again | 11 (16.9) | 6 (17.1) |  |
| Referral with persistent symptoms | 51 (78.5) | 28 (80.0) |  |
| **Symptoms and signs at illness onset** |  |  |  |
| Fever | 51 (78.5) | 24 (68.6) | 0.276 |
| Shortness of breath | 28 (43.1) | 19 (54.3) | 0.284 |
| Dyspnea | 5 (7.7) | 4 (11.4) | 0.716 |
| Chest tightness | 11 (16.9) | 8 (22.9) | 0.471 |
| Myalgia | 13 (20.0) | 7 (20.0) | 1.000 |
| Dry cough | 16 (24.6) | 9 (25.7) | 0.904 |
| Productive cough | 30 (46.2) | 17 (48.6) | 0.817 |
| Fatigue | 22 (33.8) | 12 (34.3) | 0.965 |
| Diarrhea | 4 (6.2) | 3 (8.6) | 0.693 |
| Headache | 3 (4.6) | 2 (5.7) | 1.000 |
| **Comorbidities** |  |  |  |
| Hypertension | 17 (26.2) | 13 (37.1) | 0.253 |
| Type II diabetes mellitus | 12 (18.5) | 8 (22.9) | 0.600 |
| Coronary artery disease | 4 (6.2) | 2 (5.7) | 1.000 |
| COPD | 3 (4.6) | 3 (8.6) | 0.420 |
| Hepatitis B | 3 (4.6) | 2 (5.7) | 1.000 |
| Malignancy | 2 (3.1) | 2 (5.7) | 0.610 |
| **Smoker** | 5 (7.7) | 2 (5.7) | 1.000 |
| **Alcohol user** | 2 (3.1) | 1 (2.9) | 1.000 |
| **Severity of COVID-19 pneumonia** |  |  | 0.166 |
| Mild | 0 | 0 |  |
| General | 51 (78.5) | 23 (65.7) |  |
| Severe | 14 (21.5) | 12 (34.3) |  |
| Critical | 0 | 0 |  |

COPD, chronic obstructive pulmonary diseases.

**Supplementary Table S2** Sensitivity analysis of clinical characteristics between patients included in regression analysis and the overall cohort

| **Clinicopathologic characteristics** | **Study cohort**  **(n=68)** | **Patients included in regression analysis (n=35)** | **p value** |
| --- | --- | --- | --- |
| **Laboratory results** |  |  |  |
| WBC # (×10^9/L) | 6.00 (5.05, 7.10) | 5.70 (5.10, 7.50) | 0.997 |
| RBC # (×10^12/L) | 4.10 (3.80, 4.45) | 4.04 (3.74, 4.37) | 0.295 |
| Platelet # (×10^9/L) | 230.00 (184.5, 291.50) | 220.00 (177.00,260.00) | 0.402 |
| NLR | 2.35 (1.77, 3.45) | 2.44 (1.75, 3.16) | 0.988 |
| C-reactive protein (mg/L) | 2.90 (1.59, 9.66) | 2.40 (1.36, 6.28) | 0.329 |
| Normal | 35 (53.8) | 24 (68.6) |  |
| Increased | 30 (46.2) | 11 (31.4) |  |
| Procalcitonin (ng/ml) (n=38) | 0.05 (0.03, 0.06) | 0.05 (0.03, 0.065) | 0.468 |
| Normal | 27 (71.1) | 16 (64.0) |  |
| Increased | 11 (28.9) | 9 (36.0) |  |
| Albumin (g/L) | 38.15 (35.35, 40.65) | 37.80 (34.40, 40.70) | 0.840 |
| ALT (IU/L) | 30.15 (16.60, 49.85) | 29.50 (15.10, 37.80) | 0.546 |
| AST (IU/L) | 21.80 (17.25, 29.20) | 20.90 (15.40, 25.90) | 0.515 |
| HBDH (IU/L) | 152.90 (128.20, 193.43) | 148.50 (127.60, 194.30) | 0.916 |
| LDH (IU/L) | 181.15 (156.83, 239.95) | 175.00 (156.60, 246.50) | 0.899 |
| CK (IU/L) | 48.05 (34.35, 70.43) | 44.90 (32.50, 70.80) | 0.925 |
| CK-MB (IU/L) | 7.75 (6.40, 11.50) | 8.20 (6.80, 10.60) | 0.728 |
| SARS-CoV-2 IgM (AU/ml) (n=35) | 39.01 (17.74, 90.51) | 39.01 (17.74, 90.51) | 1.000 |
| SARS-CoV-2 IgG (AU/ml) (n=35) | 142.09 (116.40,179.86) | 142.09 (116.40,179.86) | 1.000 |
| **Treatment details** |  |  |  |
| Antivirals | 26 (40.0) | 11 (31.4) | 0.424 |
| Antibiotics | 37 (56.9) | 17 (48.6) | 0.397 |
| Traditional Chinese medicine | 62 (95.4) | 34 (97.1) | 0.870 |
| Corticosteroid | 12 (18.5) | 6 (17.1) | 0.669 |
| Hypnotics | 26(40.0) | 14 (40.0) | 1.000 |

ALT, Alanine aminotransferase; AST, Aspartate aminotransferase; HBDH,α-Hydroxybutyrate dehydrogenase; LDH, lactase dehydrogenase; CK, creatine kinase; CK-MB, creatine kinase isoenzyme.
